# SIRT6 Through the Brain Evolution, Development, and Aging

**DOI:** 10.3389/fnagi.2021.747989

**Published:** 2021-10-13

**Authors:** Alfredo Garcia-Venzor, Debra Toiber

**Affiliations:** ^1^Department of Life Sciences, Ben-Gurion University of the Negev, Beer Sheva, Israel; ^2^The Zlotowski Center for Neuroscience, Ben-Gurion University of the Negev, Beer Sheva, Israel

**Keywords:** Sirtuin 6, Sirtuins, brain development, neurogenesis, brain aging, neurodegeneration, epigenetics, DNA damage

## Abstract

During an organism’s lifespan, two main phenomena are critical for the organism’s survival. These are (1) a proper embryonic development, which permits the new organism to function with high fitness, grow and reproduce, and (2) the aging process, which will progressively undermine its competence and fitness for survival, leading to its death. Interestingly these processes present various similarities at the molecular level. Notably, as organisms became more complex, regulation of these processes became coordinated by the brain, and failure in brain activity is detrimental in both development and aging. One of the critical processes regulating brain health is the capacity to keep its genomic integrity and epigenetic regulation—deficiency in DNA repair results in neurodevelopmental and neurodegenerative diseases. As the brain becomes more complex, this effect becomes more evident. In this perspective, we will analyze how the brain evolved and became critical for human survival and the role Sirt6 plays in brain health. Sirt6 belongs to the Sirtuin family of histone deacetylases that control several cellular processes; among them, Sirt6 has been associated with the proper embryonic development and is associated with the aging process. In humans, Sirt6 has a pivotal role during brain aging, and its loss of function is correlated with the appearance of neurodegenerative diseases such as Alzheimer’s disease. However, Sirt6 roles during brain development and aging, especially the last one, are not observed in all species. It appears that during the brain organ evolution, Sirt6 has gained more relevance as the brain becomes bigger and more complex, observing the most detrimental effect in the brains of *Homo sapiens*. In this perspective, we part from the evolution of the brain in metazoans, the biological similarities between brain development and aging, and the relevant functions of Sirt6 in these similar phenomena to conclude with the evidence suggesting a more relevant role of Sirt6 gained in the brain evolution.

## Evolution of the Brain

**FIGURE 1:**
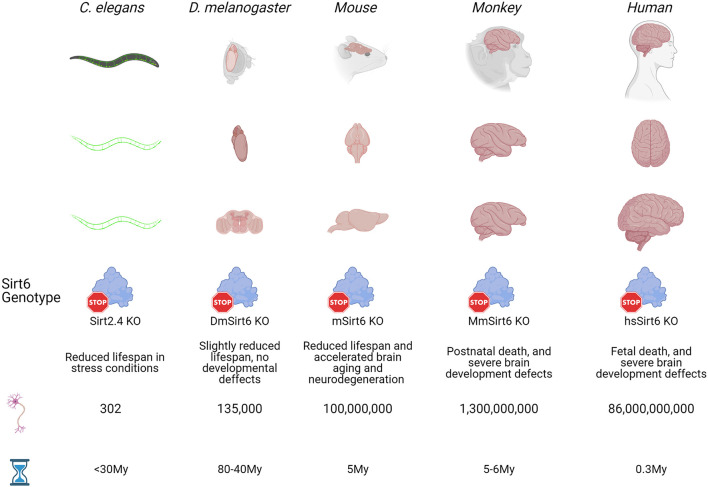
Evolutionary time of model organisms and Sirt6 function. Five well studied organisms (*C. elegans, D. melanogaster*, mouse, monkey and human) that appeared at different times during evolution are used here to show how during evolution the brains has become bigger and more complex, with an expected increase in the number of neurons. Also, as brains becomes bigger and the number of neurons increase, the effect of Sirt6 deletion on organism development and life expectancy is more evident. From slight effects on life span from relatively simple organism like worms and flies, to phenotypes with severe neurodevelopmental defects that are incompatible with life in primates. Sirt2.4 is the Sirt6 ortholog in *C. elegans*, DmSirt6 is the *D. melanogaster* Sirt6 ortholog, mSirt6 is the mouse Sirt6 ortholog, MmSirt6 is *M. Mulatta* ortholog, and hsSirt6 is the human protein.

The central nervous system (CNS) genealogy can be tracked down to the ancestral CNS or nerve cord of the common ancestor Urbilateria. During brain evolution, its complexity has changed dramatically. For example, in simpler organisms, the CNS consists of a small subset of neurons, 302 nerve cells in the nematode *Caenorhabditis elegans*, or the relatively simple *Drosophila melanogaster* brain composed of two neural ganglia (supraesophageal and subesophageal) (Hirth and Reichert, 1999; Zheng et al., 2018). Further in evolution, more complex brains appeared with more structures to process a higher quantity of environmental information and specialized motor activity. Finally, some of the neural populations observed in reptile and bird brains gave rise to the neocortex. Last, an anatomical structure with a laminar neuron array unique to mammals is the base of the mammalian complex cognitive behaviors. Mammals, birds, and reptiles originate from a common ancestor that lived 320 million years ago, believed to possess an ancestral cortex with three layers (Naumann et al., 2015). Even with its recent appearance and controversial phylogeny, it is clear that the mammal neocortex has continued evolving, increasing its size and mass and enriching in this way the cognitive capabilities of animals (Jarvis et al., 2005). For example, gyrification (the process by which brains acquire their characteristic folds), a phenomenon observed mainly in primates and some other mammals, drove to a surprising increase in the brain cortex without affecting the skull’s volume. This is clear when comparing the 10 million neurons of Mus musculus with the 100 billion neurons of human brains (Kaas, 2005). During the primate evolution, from prosimians to hominids, the overall brain structure was maintained; however, the size, the number of the nucleus, the number of neurons, and their connections increased (DeFelipe, 2011; Hofman, 2014). This increase in the brain and neocortex size explains the *Homo sapiens* outstanding cognitive and social characteristics. Notably, some of the human-specific genes can partially explain the increased neuron number and the expanded neo-cortex. One example is the human-specific ARHGAP11B protein. When expressed in mouse ferret or the ancestral primate marmoset (two distant mammals), it increased the proliferation of neural progenitor cells and the neocortex expansion. It also induced a certain degree of cortex gyrification in marmoset, absent in this species (Heide et al., 2020). Importantly, the brain size and neocortex increase observed in *Homo sapiens* requires a great proliferative burst in neuroprogenitor cells (NPCs) to ensure the production of the 100 billion neurons and glial cells that compose the human brain (Karten, 1997; Hevner, 2020). Figure 1 resumes the evolutionary time of different model organisms and the increase in the neuron number.

The increase in metabolic and proliferative activities in the larger brains leads to increased DNA damage, making DNA damage repair an essential mechanism for correct brain development (Di Micco et al., 2006; Bello et al., 2008). This is evidenced by the significant amount of DNA repair proteins whose function is indispensable for proper neurogenesis, like MCPH1, TopBP1, ATM, DNA Ligase IV, DNA-PKcs, BRCA1, and Sirt6 (Lee et al., 2000, 2012; Frappart et al., 2007; Liu et al., 2016; Enriquez-Rios et al., 2017; Onn et al., 2020).

In addition, DNA repair has essential role in protecting the brain from neurodegeneration. DNA damage accumulates during aging; this is more significant in brains affected by neurodegenerative diseases such as Alzheimer’s Disease (AD), Parkinson’s disease (PD), and Amyotrophic lateral sclerosis (ALS) (Lin et al., 2020; Gonzalez-Hunt and Sanders, 2021). Interestingly, most mammalian species do not develop neurodegenerative diseases as they age. This could also be attributed to the extremely long life humans can achieve and the problems that originated during the rapid development of the brain, rendering it susceptible to DNA damage accumulation. To understand this, we will explain the development of the brain and its correlations with the physiological process of brain aging.

## From Development to Aging

The mammal brain development begins early during embryo life and continues even after birth. The morphogenetic processes give rise to the anatomical nervous structures and the differentiated adult brain populations like neurons and glia. Some of these biological processes are mirrored during brain aging, although in reverse order. Reactivation of embryonic patterns in aged neurons impairs neuronal function.

During brain development, the neural stem cells (NSCs) in the ventricular zone divide constantly to generate the progenitors that will differentiate into neurons and the different glial populations. NSC is only localized in the olfactory bulb and the hippocampal ventricular zones in the adult brain (in mice), maintaining neurogenesis in these regions (Gage, 2002; Jin, 2016). However, in aged brains, the NSC in these regions becomes exhausted, causing cognitive and olfactory deficits (Jin et al., 2003; Jinno, 2011). During the late phases of brain development, the new neurons develop their connections and maturate them to form the brain circuits that will function after birth. During aging, there is a reduction in GABAergic signaling and GABA-A receptors expression, resulting in excitatory imbalances and glutamatergic hyperactivity (Heise et al., 2013; Richardson et al., 2013). In normal neural development, myelination occurs at the final stages, strengthening the connections between different brain areas. In contrast, during aging, the myelination and connections between cerebral zones are lost, which is more evident in individuals with cognitive deficits or neurodegeneration (Jakovcevski et al., 2009; Kohama et al., 2012).

Embryonic neurogenesis relies heavily on mitochondrial function for proper differentiation and function of new neurons. During neurogenesis, the differentiation of NSC to neurons is accompanied by an increase in mitochondria’s number, volume, and metabolic activity. This is necessary to increase the oxidative phosphorylation rate for the highly energetic-demanding functions of neurons (Khacho and Slack, 2018). However, during aging, the mitochondria undergo changes that negatively affect their function, such as membrane fragmentation, impairment in electron transport chain, and accumulation of mtDNA damage, in part due to increased ROS production (Santos et al., 2012; Morozov et al., 2017). Noteworthy, mitochondrial dysfunction and ROS increase are among the causes of the NSC exhaustion and hallmarks of neuronal aging. Furthermore, the reduced antioxidant defenses have been recognized as one of the main mechanisms that impair neuronal function by increasing the mutation burden and the quantity of misfolded non-functional proteins (Labuschagne et al., 2013; Stefanatos and Sanz, 2018). In this sense, it has been reported that in developing brains, there are reduced ROS scavenging proteins and antioxidant molecules, which makes the brain particularly vulnerable to oxidative stress, as observed with alcohol ingestion by pregnant women (Ikonomidou and Kaindl, 2010).

### Epigenetic Regulation

Brain development and cellular differentiation are regulated by epigenetic mechanisms that allow the expression of pluripotency genes in NSCs while repressing neuronal and glial differentiation genes. When NSCs differentiate into NPCs, these epigenetic mechanisms now repress pluripotency genes and activate genes required for neuronal and glial functions.

DNA methylation is one of the main epigenetic mechanisms that repress the expression of genes at specific developmental stages; it is required at both stages in NPCs and neurons (Costello, 2003; Numata et al., 2012; Lister et al., 2013). A global increase in DNA methylation occurs early during development; however, during aging, the DNA-methylation patterns change, decreasing the methylation level in specific genes like transposable elements, which is linked with a decline in neuronal function (Wilson et al., 1987; Barbot et al., 2002; Muotri and Gage, 2006; Li et al., 2013). The H3K27 trimethylation (H3K27me3) is an epigenetic mark catalyzed by the Polycomb repressive complex 2 (PRC2) and usually precedes DNA methylation during NSC differentiation and lineage commitment. Importantly, H3K27me3 decreases during brain aging, serving as a brain aging biomarker (Gong et al., 2015). Also, H3K27 acetylation (H3K27ac) which blocks H3K27me3, increases during brain aging, and is associated with Alzheimer’s disease development (Nativio et al., 2020). Another activating mark associated with aging and neurodegenerative disorders is H3K9 acetylation (H3K9ac) (Nativio et al., 2020). H3K9 hyperacetylation is required for the proper differentiation of NSC into neural and glial progenitors and mature neurons and glia (Večeřa et al., 2018). The increased H3K9ac occurring in aging could cause age-related NSC depletion in the adult niches. The experimental evidence accounting for the epigenetic changes occurring during brain aging suggests that this phenomenon behaves contrary to the developmental process. It is reverting from organized systems toward the unorganized state.

### DNA Damage

As stated before, the *Homo sapiens* brain has a large number of neurons in the neocortex. The neocortical neurons are produced during embryonic and fetal development in two main proliferation bursts. The initial occurs in humans at 5–7 gestation weeks and produces the neurons of the different cortex layers. The second burst occurs at 12 weeks and persists until 6 months after birth, generating late-born neuron populations and interneurons (Stiles and Jernigan, 2010). During the proliferative burst, NPCs are prone to suffer DNA damage from the oxidative damage generated by energetic metabolism or double-strand breaks (DBS) originated during replication and transcription (Vilenchik and Knudson, 2003). Importantly, higher metabolic activity in the brain creates an environment with increased byproducts, such as reactive oxygen species (ROS), that can generate DNA damage (Lothman et al., 1975; Navarro and Boveris, 2004). Besides, neurons do not divide, and adult neurogenesis is rare; thus, they need to keep DNA intact for a lifetime (Lima and Gomes-Leal, 2019). Therefore, neurodevelopmental problems can arise from DNA damage, while its gradual accumulation leads to neurodegeneration. Multiple repair pathways exist to cope with DSB, and its importance is exemplified by the neurological disorders caused by inherited mutations in DNA repair genes (Kakarougkas and Jeggo, 2014).

Diseases associated with impaired DNA repair include Ataxia Telangiectasia, Nijmegen breakage syndrome (NBS), Cokayne syndrome, among others (O’Driscoll and Jeggo, 2008). Ataxia-telangiectasia is an autosomal recessive disorder caused by the loss of the function of ATM gene. The disease is characterized by neurodegeneration, ocular telangiectasias, immunodeficiency, and cancer susceptibility (Chun and Gatti, 2004). Also, ATM loss of function can lead to abnormal neurodevelopment and microcephaly (O’Driscoll and Jeggo, 2008). NBS is also an autosomal recessive disease commonly accompanied by microcephaly and developmental retardation (Digweed and Sperling, 2004). In Cockayne syndrome, the patients present severe developmental abnormalities and dwarfism and progress through infant neurodegeneration (Kraemer et al., 2007). Microcephalin gene (MCHP1) mutations make NPCs hypersensitive to DSBs, causing increased apoptosis in the cortex and embryonic lethality due to impaired DDR. During DSB repair, MCHP1 forms discrete foci with other repair proteins like MDC1, 53BP1, ATM, ATR, RAD17, and RPA34. Its interaction with the SWI/SNF chromatin remodeling complex allows chromatin relaxation at the damage site.

Furthermore, HR repair pathway loss results in early embryonic lethality, and the loss of NHEJ factors leads to dysfunctional neurological phenotypes (Liu et al., 2016). One reason for this phenomenon is that HR is usually used by cells in late S or G2 phases. However, NHEJ is used by quiescent and non-cycling cells, and its malfunction impairs mainly differentiated cells like neurons or their progenitors. For example, loss of XRCC4 and LIG4, which works in the last steps of NHEJ, causes massive cell death of newly generated post-mitotic neurons (Gao et al., 1998). Hence, NHEJ is needed for the proper CNS development, and DNA damage, especially DSB, usually occurs during embryonic CNS and neocortex development. Also, developmental DNA damage causes genetic mosaicism in mature neurons, with an enhanced amount of aneuploidy, copy number variations (CNV), and chromosomal alterations. A recent study showed that nearly 41% of the neurons have CNVs. Most of the changes occurred in sub-chromosomal regions ranging in size from 2.9 to 75 Mb and chromosomal gains and losses. Importantly, the CNVs were not clonally originated and occurred in individual cells because the same mutation is not present in neuronal clusters (McConnell et al., 2013). However, in a second study using single-cell RNAseq, most of the cells had CNVs of at least 1 Mb, and these CNVs appeared during neurogenesis in NSCs generating clonality (Cai et al., 2014). Some scientists suggest that this somatic mosaicism may have a biological role in generating neuronal diversity, novel functions, and increasing the brain’s plasticity (Weissman and Gage, 2016). Also, this mosaicism can affect key genes regulating proliferation or migration, causing diseases like Autism spectrum disorder, schizophrenia, or epilepsy (Poduri et al., 2013; McConnell et al., 2017).

Interestingly, Neurons show recurrent DSB clusters (RDCs), genomic sites with elevated transcription and late replication time that are prone to suffer DSBs during proliferative bursts or differentiation. These RDC are repaired mainly by NHEJ and appear to be mainly localized in gene clusters associated with neuronal function and synaptogenesis (Wei et al., 2016). This phenomenon could be a physiological mechanism allowing the generation of different neural cells, with functional diversification (Wei et al., 2018). Furthermore, neuronal mosaicism is also caused by the developmental activation of retrotransposable elements, like LINE-1 (L1) elements. Evidence of retrotransposons activation is found in hippocampal and caudate nucleus neurons. The activation of these transposons occurs in NPCs during neurogenesis, generating novel genetic information and neuronal mosaicism (Baillie et al., 2011). Therefore, proper DNA repair machinery is necessary for normal brain development.

## Sirt6 Roles in DNA Repair and Epigenetic Regulation

Sirtuins are protein deacetylases that use NAD + as a cofactor, with a great diversity of biological and biochemical functions (Houtkooper et al., 2012). The first discovered Sirtuin protein was Silent information regulator 2 (Sir2) in Saccharomyces cerevisiae, an epigenetic regulator implicated in lifespan that silences telomeres and mating locus in yeast (Kaeberlein et al., 1999; Guarente, 2011). Afterward, more Sirtuins were discovered in yeast (Sir1-2) and other model organisms, including mice and humans. Mammals have seven Sirtuin proteins (Sirt1-7), with different biological functions and cellular localization, regulating gene expression and several signaling pathways (Haigis and Sinclair, 2010). Sirtuins have attracted the attention of several scientists due to their functions in lifespan regulation and aging.

Sirt1 is the family member with the closest similarity to yeast Sir2 ortholog. It has a deacetylase and deacetylase activity, and unlike Sirt6, Sirt1 shuttles from the cytoplasm and nucleus. The main cellular activities of Sirt1 are related to the deacetylation and deacylation of histones and other transcriptional regulators. Sirt1 is one major regulator of cellular metabolism and insulin signaling, mainly by the negative regulation over PGC-1α and PPARα transcription factors (Krzysiak et al., 2018). Furthermore, Sirt1 regulates different DNA repair pathways mainly by the deacetylation of repair proteins like XPA, RPA1, or BRCA1 (Zhao et al., 2017; Jarrett et al., 2018; Lahusen et al., 2018). Sirt2 is a mainly cytoplasmic protein that shuttles between nucleus and cytoplasm and controls cellular metabolism by targeting different glycolytic enzymes, aldolase, and glyceraldehyde 3-phosphate dehydrogenase, phosphoglycerate kinase, and enolase (Cha et al., 2017). Sirt3-5 are mainly located to the mitochondria with various metabolic roles. Moreover, it has been observed that after hydroxyurea-induced replication stress, Sir2 deacetylates ATR- interacting protein (ATRIP), increasing the interaction of ATR kinase with RPA in the damaged DNA (Cha et al., 2017). Sirt7 is a nuclear protein that mainly localizes in the nucleolus. It has been observed that Sirt7 is required for the proper rDNA silencing, and Sirt7 deficiency cause rDNA instability and cellular senescence (Paredes et al., 2018).

**FIGURE 2:**
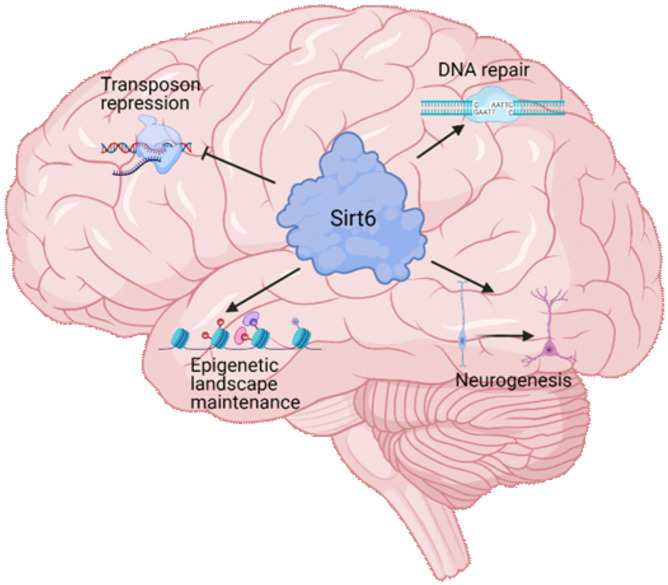
Sirt6 functions in brain physiology. Sirt6 is a multifunctional protein that regulates several cellular processes. During brain development and brain aging, Sirt6 regulates four major processes. The repression of transposable elements, which is necessary for the genome integrity maintenance. The proper function of DNA repair pathways that maintains the genome integrity to promote neuronal survival and function. The epigenetic landscape maintenance through its histone deacetylase activity that allows Sirt6 to repress the expression of specific genes and maintain neuronal identity. The neurogenesis process, which produce the mature neurons during organismal development, and is necessary in adult brains to maintain the function of specific brain areas.

Sirt6 is a mammalian Sirtuin notably related to aging and lifespan (Sirt6 targets and its mechanisms associated with aging are summarized in Table 1) (Vitiello et al., 2017). It is mainly nuclear and has different enzymatic activities, like ADP-ribosyl transferase, protein deacetylation, protein long-fatty acid deacylation, and DNA binding (broken-ended ssDNA) at DSB sites (Chang et al., 2020). Sirt6 was first described as a mono ADP-ribosyl transferase that catalyzes the mono ADP-ribosylation of itself, PARP1, and Kap1, among other proteins (Liszt et al., 2005; Mao et al., 2011; Van Meter et al., 2011). However, the most described Sirt6 function is its activity as histone (and other proteins) deacetylase (Pan et al., 2011). Through the deacetylation of the lysine 9 of the histone H3 and lysine 56 (H3K9ac, H3K56ac), Sirt6 represses the expression of its target genes and functions as a co-repressor for transcription factors like c-Myc, NF-kB, or HIF-1 (Michishita et al., 2008; Kawahara et al., 2009; Yang et al., 2009; Zhong et al., 2010). Furthermore, during mitosis, Sirt6 deacetylate H3K18ac at pericentromeric regions, preventing mitotic segregation errors, genomic instability, and cellular senescence (Tasselli et al., 2016). Importantly, Sirt6 also deacetylates non-histone proteins, like PKM2, SMAD3, NAMPT, and p27, all of them implicated in several essential cellular processes like metabolism, cell differentiation, and cellular senescence (Bhardwaj and Das, 2016; Zhao et al., 2016; Sociali et al., 2019; Zhong et al., 2020). Finally, Sirt6 can remove long-fatty acid (deacylation) (Jiang et al., 2013). Also, Sirt6 activity can be enhanced by long-chain fatty acids (Wang W. W. et al., 2016). The deacylase function is also important for the proper secretion of proteins such as TNF-α or to counteract the activity of membrane-targeted proteins such as R-Ras2, which needs to be myristoylated for proper membrane localization (Feldman et al., 2013). In Figure 2, we summarize the main functions of Sirt6 during brain development and aging.

**TABLE 1:** Sirt6 molecular targets associated with the aging process.

Sirt6 target	Cellular function related to aging	Organism	References
H3K9ac	The aging process in cardiomyocytes reduced Sirt6 levels, and increase H3K9ac, one of its canonical substrates. This phenomenon is associated with the increased expression of IL-6 and p21, which induce a senescence phenotype in cardiomyocytes. Also, the antiaging flavone acacetin counteracts age-related cardiomyocyte senescence by Sirt6 expression and the subsequent decrease in H3K9 acetylation	Mice	Hong et al., 2021; Pillai et al., 2021
Nrf2	In cardiomyocytes, Sirt6 activates Nrf2 signaling and Sirt3 expression by two mechanisms; inhibiting KeapI expression, a negative regulator of Nrf2; Direct binding to Nrf2 and inhibition of KeapI binding. This mechanism protects hearts from developing diabetic cardiopathy. Also, Sirt6 and Nrf2 functional interaction is associated with the alleviation of the pro-inflammatory microenvironment observed in atherosclerotic endothelium.	Mice	Kanwal et al., 2019; He et al., 2021
Gluconeogenesis genes	In transgenic mice overexpressing Sirt6, it was observed a direct increase in the expression of Pck1, Pcx, G6pc, and Fbp1. Which resulted in increased gluconeogenesis, better energetic metabolism homeostasis, and finally in a lifespan and well-being increase.	Mice	Roichman et al., 2021
TFAM	In aging Polyandrocarpa misakiensis (tunicate) is observed a reduction in mitochondrial activity, due to reduced expression of mitochondrial transcription factor TFAM. The interaction of Sirt6 with the transcription factor YY1 and its coregulator YAF2 allows Sirt6 to deacetylate (H3K9ac) the promoter of TFAM, repressing its expression.	Budding tunicantes	Kawamura et al., 2021
YY1	Sirt6 interacts with YY1 and together they regulate the expression of several genes implicated in RNA splicing, proteosome catabolism, cell cycle transition, and chromosome organization. Interestingly, during brain aging, the decreased expression of Sirt6 impairs YY1 function affecting these cellular processes and initiating pathological aging in the brain.	Mice	Stein et al., 2021
NF-κβ	Angiotensin II signaling induce vascular aging in the aortae of rats. This mechanism is in part the result of a decreased Sirt6 expression and increased NF-κβ activity. Sirt6 interacts with NF-κβ, and deacetylates NF-κβ K310, reducing the transcriptional activity of this TF. Also, caloric restriction induces Sirt6 expression, causing the deacetylation of NF-κβ K310, and attenuating NF-κβ signaling. Also, Sirt6 inhibits NF-κβ binding to Myostatin promoter, inhibiting age-related muscle dysfunction.	Rat/Mice	Samant et al., 2017; Liu et al., 2021
Prx1, Srx, and Txnip	In chondrocytes from old subjects, an increased level of ROS and oxidative stress is found. Mainly through the decrease in the activity of Sirt6, the decreased expression of antioxidant proteins Prx1 and Srx, and the increase in the antioxidant inhibitor Txnip. Sirt6 overexpression decreases oxidative stress by the expression of Prx1, and repression of Txnip	Human	Zhang et al., 2016; Collins et al., 2021
hTERT	In cells from hypertrophic ligamentum flavum, observed in aged individuals with lumbar spinal stenosis, it is observed a reduction of Sirt6 expression. Sirt6 overexpression increased the levels and activity of hTERT, reducing the number of cells that present a senescent phenotype.	Human	Chen et al., 2021
HIF-1α	In atherosclerotic plaques, Sirt6 interacts with HIF-1α inhibiting its ubiquitination and posterior degradations. HIF1α stabilization leads to angiogenesis in endothelial cells.	Mice	Yang et al., 2021
FOXM1	Sirt6 regulates the expression of the transcription factor FOXM1. vascular aging, cause the reduction of Sirt6 and the subsequent repression of FOXM1. FOXM1 expression in Sirt6 KO vascular cells promoted cell cycle progression and ameliorates senescence.	Human	Lee et al., 2020
Hus1 and MYH	Sirt6 acts as an early sensor of oxidative DNA damage in telomeres, recruiting Hus1 from the Rad9-Rad1-Hus1 complex, and MYH glycosylase. Sirt6 repress telomere attrition cellular senescence by improving DNA repair in the telomeres.	Human	Tan et al., 2020
Cathepsin B	CatB leakage from the lysosome is found in the microglia of the brain cortex in old mice and associates with age-related cognitive impairment. CatB degrades Sirt1, Sirt6, and Sirt7 after its nucleus translocation, and promotes brain aging.	Mice	Meng et al., 2020
Tau	Tau is a cytoskeletal protein, which abnormal function and aggregation are associated with age neurodegeneration like Alzheimer’s disease. The acetylation of Tau in the lysine 174, cause its nuclear transport and activation of the expression of rDNA genes, ultimately depleting the energy from the cells and causing neuronal death. Sirt6 deacetylates TauK174ac blocking its nuclear import. However, under DNA damage or Sirt6 age-associated decrease, the acetylation of Tau increases promoting neurodegeneration. Also, Tau is stabilized by phosphorylation, causing pathological accumulation of the protein. GSK3 is one kinase that phosphorylates Tau, and this phosphorylation is inhibited by Sirt6. Under Sirt6 absence, Tau is hyperphosphorylated accumulating in neurons.	Human/Mice	Kaluski et al., 2017; Portillo et al., 2021
PCSK9	PCSK9 is a crucial regulatory protein for LDL-cholesterol metabolism by inducing LDL receptor degradation. Sirt6 interacts with the TF FOXO3 in the promoter of the PCSK9 gene, deacetylating H3K9ac and H3K56ac, and causing its repression. Sirt6 overexpression in fat mice, cause LDL-cholesterol levels to decrease.	Mice	Tao et al., 2013
DNA double-strand breaks	DNA damage, specially DSB is associated with aging and pathological aging. Sirt6 can bind directly to broken DNA, and initiate the DSB repair pathway by the recruitment of other repair proteins such as ATM, PARP1, Mre11, and Ku80/70.	Human/Mice	Onn et al., 2020
MALAT1	Endothelial-to-Mesenchymal transition is a morphogenetic process observed in aged vascular cells. Sirt6 binds to the MALAT1 promoter repressing its expression and inhibiting the initiation of the EMT process.	Mice	Qin et al., 2019
Lamin A	Lamin A is a regulator of Sirt6 functions in DSBs repair. Lamin A interacts with Sirt6, activating it and facilitating its localization in damaged DNA. Lamin A promotes Sirt6-mediated recruitment of DNA-PK, CtIP deacetylation, and PARP1 poly-ADP ribosylation. Laminopaties, like Hutchinson-Gilford progeria syndrome, may impair Sirt6 function, and induce aging by DNA damage accumulation.	Human	Ghosh et al., 2015
PARP1	Sirt6 poly-ADP rybosylates PARP1 activating it and promoting its poly-ADP rybosylase activity. The activation of PARP1 by Sirt6 can alleviate the age-related decline in the BER repair mechanism. Importantly, this ADP ribosylation of PARP1 appears to be highly correlated with the lifespan of mammals, and especially by the repair of DSB DNA damage.	Human	Xu et al., 2015; Tian et al., 2019
p53	Sirt6 interacts and deacetylates p53 in K381, inducing its degradation. It has been observed that p53 haploinsufficiency increases the lifespan, and ameliorates the aging phenotype of Sirt6 KO mice.	Mice	Ghosh et al., 2018
p27	Sirt6 represses the expression of p27 and deacetylates the protein inducing its degradation. This allows Sirt6 to counteract the senescence phenotype.	Human/Mice	Zhao et al., 2016
SMAD3	Fibrosis is a common trait of the aging phenotype and is caused by the increase in the myofibroblast population. Sirt6 negatively regulates myofibroblasts generation by deacetylating H3K9ac and H3K56ac in the promoter of SMAD3. Repressing SMAD3 transcription attenuates the response of fibroblasts to TGF-β and reduces the fibrotic processes.	Human/Mice	Maity et al., 2020
ERK1/2	Sirt6 regulates negatively the expression of ERK1/2 genes, by the deacetylation of H3K9ac and H3K56ac in its promoters. ERK1/2 repression by Sirt6 alleviates renal injury by cisplatin.	Mice	Li et al., 2018
Wnt	Sirt6 interacts with the LEF1 transcription factor and inhibits the expression of several Wnt target genes by the deacetylation of H3K56ac in the promoters. This mechanism allows Sirt6 to control the self-renewal and homeostasis of hematopoietic stem cells.	Mice	Wang H. et al., 2016
Notch	In different kidney diseases, podocytes injury is a phenomenon that precedes renal dysfunction. It has been demonstrated that Sirt6 protects podocytes from injury by the deacetylation of H3K9ac in the promoters of Notch1/4.	Mice/Human	Liu et al., 2017

## Sirt6 in Neurogenesis

Sirt6 is highly expressed in mature neurons, and it is vital for the maintenance of genomic stability, gene regulation, and metabolism. However, little is known about Sirt6 functions during neurogenesis and in NSCs or NPCs. During development, Sirt6 represses the expression of pluripotent transcription factors Oct4, Sox2, and Nanog in ESCs differentiation, and Sirt6 deficiency increases the efficiency of somatic cell reprogramming, suggesting Sirt6 is a regulator of pluripotency. Importantly, the differentiation of Sirt6 KO ESCs was abnormally directed to the neuroectoderm lineage, a phenotype resulting from Tet1/2 derepression, which demethylates the promoters of neural differentiation regulating genes (Etchegaray et al., 2015). Moreover, Sirtuins 1,2, and 6 are needed for post-ischemic neurogenesis in mice. Interestingly, Sirt1/2 increased NSCs survival and proliferation, but Sirt6 was required for proper NSCs differentiation into neurons (Zhao et al., 2015). During adult neurogenesis, Sirt6 overexpression in mice increased hippocampal neurogenesis, by the increment in young neuron production, without affecting the NSC pool and glial cell production (Okun et al., 2017). Also, the progressive reduction of Sirt6 expression during aging suggests that Sirt6 absence impairs the proper differentiation of neurons and NSCs pool homeostasis in aged brains.

Acetylated H3K56, a Sirt6 substrate, cooperates with the histone variant H2AZ.1 to regulate the differentiation of NPCs into glial cells. Thus, H3K56ac or H2AZ.1 reduction abrogates gliogenesis and impairs the mouse brain’s normal development (Su et al., 2018). H3K56ac is also associated with pluripotency maintenance in ESCs since it is required for strong Oct4 binding to its targets (Tan et al., 2013). Hence, the H3K56 deacetylation by Sirt6 may have important roles during gliogenesis or NSCs pool maintenance.

Acetylated H3K9 is another transcription activation mark targeted by Sirt6, especially in enhancer regions. In Drosophila, H3K9ac along with H3K27me3 (a repressive mark) regulates NSCs differentiation to neural and glial progenitors and lastly to neurons and glial cells. H3K9ac appears to be highly enriched in NSCs, but its global quantity decreases toward neural and glial differentiation, with specific enrichment in neuronal genes, activating lineage-specific gene expression (Chen X. et al., 2019). H3K9ac is also important for c-Myc activation of pluripotency genes in mouse ESCs, and it decreases as cells progress through differentiation (Hezroni et al., 2011; Seruggia et al., 2019). This evidence implies that H3K9ac maintains euchromatin and preserves the pluripotency state of ESCs. Sirt6 role in H3K9ac deacetylation has not been addressed during development.

Nevertheless, like other histone deacetylases, Sirt6 could be regulating H3K9ac levels in specific promoters to drive proper NSCs differentiation into mature lineages. For example, the increased risk of neural tube defects in diabetic pregnant women is in part a consequence of a decrease in the expression of Sirt6 and Sirt2 due to high glucose concentrations. Sirt6 decrease leads to aberrant epigenetic regulation (including aberrant H3K56ac and H3K9ac deposition), which causes defects in neural tube closure (Yu et al., 2016).

The decreased neurogenesis in the hippocampus subventricular zone is one of the reasons for the age-related declined cognitive abilities and dysfunctions. In aging, the brain regions where neurogenesis occurs slowly exhaust NSCs pools. However, Sirt6 role in age-related decreased neurogenesis is still unclear. Sirt6 overexpression in the hippocampus of aged mice increases the number of new neurons without changing the number of new glial cells and the proliferation of NSCs (Okun et al., 2017). This highlights parallelism in Sirt6 function between adult and developmental neurogenesis, which controls neurons’ proper differentiation. This was also observed in a model of regenerative neurogenesis after ischemic strokes in rats, where NAD + treatment increased regenerative neurogenesis, and its effect was dependent on Sirt1 for NSC proliferation and self-renewal, and in Sirt6 and Sirt2 for proper neuronal and glial differentiation (Zhao et al., 2015).

Interestingly, since Sirt6 is necessary for the proper NSC differentiation into mature neurons, the age-related decreased expression of Sirt6 could impair the regenerative capabilities of adult NSCs. Also, the Sirt6 is a DSB sensor protein and regulator of repair pathways. Therefore, its absence can impair the ability of NSCs to cope with DNA damage, accumulate genomic alterations, and depleting the NSC pool.

**FIGURE 3:**
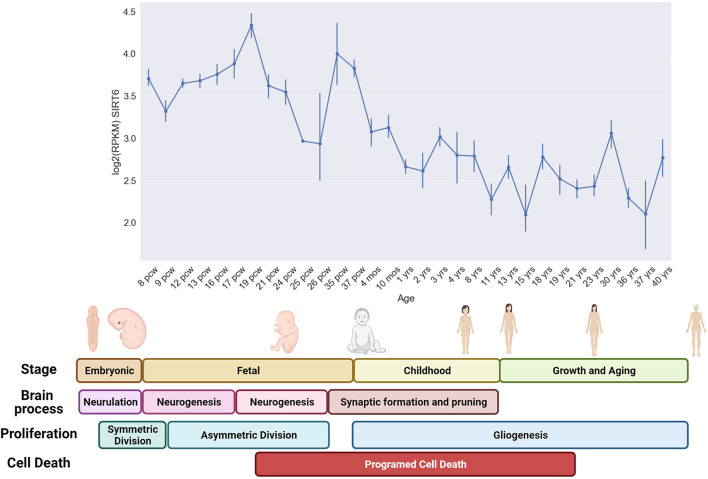
Sirt6 expression during human development and life. In the upper graph, we show the mean expression level [Log2 (FPKM)] of several brain regions during specific developmental time points (Hawrylycz et al., 2012; Miller et al., 2014). The expression level was measured from post-conceptional week 8–9 (PCW) which correspond to the end of the embryonic stage and the start of the fetal stage (that last until birth). The formation of the primitive neural tube with the embryonic pouches that will give rise to the different CNS regions occurs during embryonic stages (Neurulation). At the beginning of the fetal period (6 pcw), the neural stem cells start to proliferate mainly through symmetric division that generates two NSCs. This symmetric division progressively switches toward an asymmetric division that generates a neural progenitor and a differentiated neuron, this shifting occurs approximately at 10 pcw and lasts until neurogenesis is completed at 27 pcw. Early during neurogenesis, the formation of synaptic connections occurs, and lasts until childhood, also programmed cell death is visible in neurogenic regions since the beginning of this process. However, programmed cell death occurs mainly in perinatal stages, and allows the survival of only those neurons that were capable to form stable and functional connections. Gliogenesis occurs mainly in postnatal periods. The expression of Sirt6 shows an increase that coincides with the proliferative burst of asymmetric division of neural progenitors and decreases toward the period when programmed cell death begins to clean those non-functional neurons (Stiles and Jernigan, 2010).

Interestingly, analyzing the expression of Sirt6 during fetal, childhood, and adult life, we observed that the brain expression of Sirt6 shows peaks of expression, especially during fetal development. Interestingly, the first Sirt6 expression peak starts to increase when NPCs change their division mechanism from symmetrical to asymmetrical division and starts to decline toward the end of the first proliferative burst. This change is important since non-differentiated cells like NPCs or NSCs have two division mechanisms. The symmetrical division allows the maintenance of the progenitor’s pool by generating two daughter cells with the same phenotype. The asymmetrical division correlates with the differentiation process since it generates one daughter cell that commits into differentiation and one daughter cell that maintain the progenitor phenotype. These peaks are present at the moments of increased proliferation, like during neurogenesis and gliogenesis but decrease when programmed cell death appears (Figure 3). These observations strengthen the hypothesis that Sirt6 is necessary for leaving a pluripotent stem state toward a differentiated neuronal state and is required during proliferative phases to cope with increased DNA damage.

## Sirt6 During Aging

### DNA-Damage

The cellular genome is constantly threatened by endogenous and exogenous factors that can damage it, altering its structure and sequence and ultimately leading to aging and disease. One of the most accepted biological aging theories states that the DDR mechanisms performance decrease and DNA damage is accumulated, leading to progressive aging. Sirt6 is one of the main regulators of various DNA repair pathways. Sirt6 is a DSB sensor that initiates the DNA damage response. It localizes at damaged sites and cooperates with other repair proteins to restore DNA integrity. Interestingly, Sirt6 knock-out in mice has an accelerated aging-like phenotype, and cells tend to accumulate DNA damage (Mostoslavsky et al., 2006). Sirt6 availability decrease is associated with aging in different tissues, while Sirt6 overexpression extends mice lifespan, and one of the crossroads between Sirt6 function and aging is its role in DNA repair.

Another pathway affected by Sirt6 is BER, in which lack of Sirt6 weakens BER repair, and its overexpression rescues the age-related decrease in BER repair efficiency (Mostoslavsky et al., 2006; Xu et al., 2015). In abasic damaged DNA sites, Sirt6 induces the recruitment of PARP1, which is required for the recruitment of XRCC1 (Okun et al., 2017). Furthermore, under oxidative DNA lesions, Sirt6 interacts with the MYH glycosylase and form a complex with APE1 nuclease and the checkpoint clamp (Rad1-Hus1-Rad9). These interactions increase MYH activity. The increased MYH activity is independent of the Sirt6 deacetylase function (Mostoslavsky et al., 2006; Hwang et al., 2015). Also, after oxidative DNA damage, Sirt6 mono-ADP-rybosilates PARP1 in K521 residue, required for PARP1 regulation of DDR (Mao et al., 2011). Finally, Sirt6 can also regulate the NER pathway by increasing the proficiency of the global-genome pathway. Under UV irradiation, Sirt6 deacetylates the NER sensor protein DDB2 in residues K35 and K77, allowing DDB2 ubiquitination and chromatin detachment (Geng et al., 2020). These studies highlight Sirt6 functions in genome integrity and their importance for DDR proper function.

Last, DSBs are considered the most lethal DNA lesion because they can lead to sequence changes, including mutations, copy number variations, chromosomic translocations, and deletions, which direct to cellular transformation, senescence, or apoptosis. DBS arises from ionizing radiation exposure, oxidative stress, replicative stress, and complications in other repair pathways. Cells have two main repair mechanisms HR and NHEJ. Importantly, in mammals, DSB repair efficiency is highly correlated with lifespan, and the long-lived species possess more efficient DSB repair. Interestingly, a high efficient DSB repair is correlated with more efficient Sirt6 activity during DSB repair, especially with its ADP-ribosylation activity (Tian et al., 2019). Highlighting the relevance of Sirt6 in DSB repair and its role in biological aging.

Sirt6 regulates several steps of the DSB repair pathways. First, Sirt6 binds ssDNA in the DSB sites and functions as a sensor that initiates the repair pathway. This binding is independent of other known DSB sensors such as PARP, Mre11, or Ku80, and it triggers ATM recruitment and γH2AX deposition. Moreover, Sirt6 absence impairs the localization and function of other repair proteins from both pathways (Onn et al., 2020). Sirt6 also regulates the chromatin compaction at DSB sites by directly deacetylates H3K56ac and H3K9ac to rearrange chromatin (McCord et al., 2009; Toiber et al., 2013). It also recruits the chromatin remodeler SNFH2, displacing nucleosomes at the damaged regions (Toiber et al., 2013). Trim66, a Bromo domain-containing protein in ESC cells, binds specifically H3K56ac, further recruiting Sirt6 to DSB sites. This recruitment causes H3K56ac deacetylation and is fundamental for maintaining the genomic integrity of ESCs and blastocysts (Chen J. et al., 2019). Sirt6 regulates the mono-ADP-ribosylation of the histone demethylase KDM2A, displaces this enzyme from chromatin leading to transcription repression at the repair sites. In addition, Sirt6 recruits CHD4, which displaces HP1 from chromatin (Hou et al., 2020; Rezazadeh et al., 2020). Finally, Sirt6 binds repair factors, like DNA-PK and PARP, and facilitates the interaction of these enzymes with other NHEJ factors like Ku80 (McCord et al., 2009; Van Meter et al., 2011; Chen et al., 2017).

Interestingly, induced pluripotent stem cells (iPSC) derived from old mice are less efficient in DSB repair than those derived from young mice, specifically in the NHEJ pathway, and Sirt6 downregulation is one of the causes of this phenomenon (Chen et al., 2017). Also, Sirt6 DSB repair functions become relevant in neurodegenerative diseases. For example, brain-specific Sirt6 knock-out mice show signs of early brain aging, like behavioral and major learning impairment and the stabilization of Tau protein, which are characteristics of different neurodegenerative diseases (Kaluski et al., 2017). Portillo et al. (2021) Furthermore, in mouse models of Alzheimer’s disease (AD), restoring Sirt6 expression prevents the accumulation of Aβ plaques in the brain in a p53-dependent manner. These results state the importance of Sirt6 in brain aging and the correlation of this biological phenomenon with defective DNA repair.

### Histones Modification

Gene expression and chromatin stability are highly regulated through chromatin remodeling and epigenetic modifications. Here, we will describe the main targets of Sirt6 relevance in DNA repair and brain function.

### H3K9ac

One of the first described Sirt6 targets was its role at telomere heterochromatin through H3K9ac deacetylation. The WRN requires this modification to keep telomeres packaging. Sirt6 knockdown cause chromosome fusions and cellular senescence (Michishita et al., 2008). Moreover, aged human brains show an increase in the acetylation levels of H3K9 and H3K27, but it is much more pronounced in Alzheimer’s disease brains. This may be due to increased activity p300, CBP, and TRAAP acetyltransferases (Braidy et al., 2015) and decreased Sirt6 and NAD + levels (Dong et al., 2019).

Furthermore, in various Tauopathies, H3K9ac is affected near the nuclear lamina (Klein et al., 2019). Moreover, in rats, H3K9ac increased in an age-dependent manner in specific promoters like BDNF and c-Fos. On the contrary decrease of H3K9ac improved memory and behavior in old rats (de Meireles et al., 2019). The abnormal increase in H3K9ac in AD brains may result from the decreased activity of Sirt6.

### H3K56ac

Sirt6 deacetylation of H3K56ac has been related to DNA damage repair, genome stability, and gene expression (Yuan et al., 2009). In the telomeres, Sirt6-mediated H3K56ac deacetylation maintains the heterochromatic state in sub-telomeric regions (Michishita et al., 2009; Tennen et al., 2011). The deacetylation of H3K56ac by Sirt6 has been associated with the regulation of glucose metabolism. In the retina, Sirt6 represses glycolysis-associated genes such as GLUT1 by deacetylating H3K56ac (Silberman et al., 2014). Also, increased H3K56ac has been associated with tumorigenic phenotypes in breast and lung cancers (Das et al., 2009; Zhu et al., 2018).

### H3K18ac

H3K18ac is an epigenetic mark absent in pericentromeric chromatin, and its absence is important to repress transcription in these regions during mitosis. HDAC’s activity deficit, like Sirt6 inhibition, cause increased transcription in pericentromeric regions and mitotic defects and genomic instability (Tasselli et al., 2016). In human cell lines, the inhibition of histone acetyltransferases (HATs) such as Gcn5 and Noggin reduced the proportion of senescent cells and increased their proliferation under culture. This effect was dependent on H3K9ac and H3K18ac (Huang et al., 2020). Other Sirtuins can also catalyze the deacetylation of H3K18. For example, Sirt7 regulates mitochondrial biogenesis, L1 transposon silencing, and DSB repair by H3K18 deacetylation (Vazquez et al., 2016; Yan et al., 2018).

### Transcription Factors

As an epigenetic regulator, Sirt6 can modulate the function of different transcription factors, operating as a co-repressor. Sirt6 negatively regulates the effect of the c-Myc transcription factor in ribosome synthesis by deacetylating H3K9ac in the promoters of ribosomal genes (Sebastián et al., 2012). Also, through H3K9ac deacetylation, Sirt6 contributes to terminate NF-kB function, preventing apoptosis. Under hypoxic and hypoglycemic stress, Sirt6 acts as a repressor of glycolytic genes preventing the metabolic shift toward glycolytic metabolism. Sirt6 interacts with HIF-1α to repress glycolytic genes by the deacetylation of H3K9ac in their promoters. Also, it blocks promoters at a poised state (Zhong et al., 2010). Overall, Sirt6 may have roles regulating brain metabolism, but specific interactions with TF involved in brain development and maintenance have not been described yet. SREBP1/2 is a transcription factor that regulates lipid metabolism in different tissues of the body and is also implicated in circadian metabolic regulation. Sirt6 negatively regulates SREPB1/2, to maintain lipid homeostasis (Elhanati et al., 2013). Furthermore, Sirt6 regulates the cyclic activity of the circadian TFs Clock and BMAL1, and couples the lipid metabolism with circadian cycles through SREPBP1 inhibition (Masri et al., 2014).

### Transposable Elements

The transposable elements or transposons are DNA sequences that can copy themselves into new genomic regions, increasing their copy number and driving genomic diversity through evolution. Transposons are known as “jumping genes” that account for nearly 45% of the human genome and are divided into several classes based on their sequence (Hua-Van et al., 2005). The activation of these transposable elements has a positive effect by driving evolution through the duplication or modification of coding genes (Feschotte and Pritham, 2007). However, its mobilization can induce insertional mutagenesis affecting coding gene function. Also, its genomic insertion induces DBSs. Some active transposable elements move through an RNA intermediary that is later retrotranscribed and integrated into the genome (Chénais, 2013).

Among transposable elements, Long-interspersed element-1 (LINE-1 or L1) contributes to approximately 17% of the genome sequence and is the only class of transposon known to be active in humans. Importantly, L1 and other transposable elements maintained inactive through its heterochromatinization by DNA-methylation and subsequent binding of heterochromatin proteins like Mecp2, HP-1, or KAP-1 to keep genome integrity (Benard et al., 2013). However, LINE1s are active in specific Spatio-temporal patterns during embryonic development and adult neurogenesis. This process helps to generate somatic mosaicism. Interestingly, the organ with more active L1 elements is the brain. This mosaicism appears particularly important for the generation of neuronal and functional diversity and plasticity. Importantly, the new insertional sites of L1 elements in the neurons correspond to active neuronal genes, highlighting the possibility of neuronal function diversification (Coufal et al., 2009). Also, L1 activity appears to be controlled by stem phenotype regulators such as Sox2 and Wnt. Sox2 represses L1 expression in hESC and NSCs, and the shift of Sox2 activity toward Wnt transcription in NSCs activates the expression of L1 transposons (Muotri et al., 2005; Kuwabara et al., 2009; Zhang et al., 2017). Also, L1 activation appears to occur during adult neurogenesis in the hippocampus (Kuwabara et al., 2009; Muotri et al., 2009).

L1 expression and integration into novel genomic positions increases during aging in humans, rodents, and *C. elegans* (De Cecco et al., 2013b; LaRocca et al., 2020). The increased L1 activity in aged brains has several impacts on neuron function. First, the integration of L1 elements into coding genes can mutate essential genes. Secondly, the ORF2 protein encoded in L1 elements can induce DSBs. Notably, the activation of L1 and other transposons during aging is mainly due to global epigenetic changes that cause derepression (Villeponteau, 1997; De Cecco et al., 2013a). One of the epigenetic regulators responsible for the gradual activation of transposons with age is Sirt6. Sirt6 binds L1 elements and drives heterochromatin formation by the recruitment and mono-ADP-ribosylation of KAP-1 repressor, which subsequently stabilizes HP-1α (Van Meter et al., 2014). In Sirt6 knockdown mice, overactivation of L1 elements was observed, leading to genomic instability, inflammation, and aging phenotype (Simon et al., 2019). The ability of Sirt6 to repress transposons contributes to maintaining genome integrity and mitigate aging phenotypes and diseases.

## Effect of Sirt6 Loss of Function During Evolution

During evolution, the brain has continued changing, being more centralized in higher organisms. As the brains become more complex in mammals, their development requires more cellular divisions. Its function is more metabolically demandant; hence it requires the diversification and specialization of proteins that cope with the increased probability of DNA damage from internal sources. In this sense, Sirt6 has gained functional diversification, which ultimately maintains genome and epigenome integrity throughout organism life, and its dysfunction is correlated with the onset of aging in complex organisms. In Figure 2, we resume the effect of Sirt6 knock-out in model organisms and its correlation with brain size.

In yeast (lacking a brain), the mechanism by which Sir2 regulates lifespan is the silencing and recombination regulation of rDNA and the asymmetric distribution of oxidized proteins between mother and daughter cells (Sinclair and Guarente, 1997; Kaeberlein et al., 2004; Delaney et al., 2011). Moreover, ySirt2 functions in silencing telomere expression of TERRA RNA (Maicher et al., 2012; Wierman and Smith, 2014).

In organisms like *D. melanogaster* and *C. elegans*, Sirtuins overexpression increases the organism’s lifespan. In contrast, its loss reduces the beneficial effect of caloric restriction over the lifespan (Rogina and Helfand, 2004; Lee et al., 2006). In *C. elegans*, only Sir-2.1 (the analog of mammalian Sir1) has been associated with increased lifespan, but there is no clear evidence regarding the implication of Sirt-2.4 (analog of mammalian Sirt6) in lifespan (Lee et al., 2006). However, *C. elegans* Sir-2.4 enhances stress survival by regulating FOXO transcription factor DAF-16 and the formation of stress granules (Chiang et al., 2012; Jedrusik-Bode et al., 2013). Interestingly, this protein is mainly expressed in a subset of head and tail neurons, which implies a possible function of Sirt-2.4 in neuronal physiology (Chiang et al., 2012). In *D. melanogaster*, Sirt6 overexpression increases fly lifespan, partially through DNA repair mechanisms enhancement. It was shown that Sirt6-overexpressing mutants were more resistant to paraquat, a genotoxic agent (Kusama et al., 2006; Taylor et al., 2018).

Moreover, knockdown of Sirt6 in *D. melanogaster* didn’t show lethality or developmental problems (Kusama et al., 2006). Behavioral experiments carried out by our group have found that Sirt6 knockdown has detrimental effects on *D. melanogaster* behavior (unpublished data). These data support that Sirt6 has major roles in *Drosophila* brain health, suggesting neurological impairments during Sirt6 loss. However, this impairment is not as severe as the one observed in organisms with more complex CNS. In higher organisms, a proliferative burst to form the neocortex is required, and Sirt6 could have a fundamental role in dealing with DNA damage and epigenetic regulation.

In mammals, Sirt6 shows more relevant roles regulating several aspects of development and physiology. In mice, Sirt6 shows a high expression in the brain, heart, and muscle. Its overexpression in mice causes an increase in lifespan, due to a general attenuation of IGF-1 signaling, but also due to better metabolic regulation of lipid and glucose metabolisms (Liszt et al., 2005; Kanfi et al., 2012; Roichman et al., 2021). Sirt6 loss in mice causes early death by 4 weeks after birth. These mice are smaller, present lymphopenia, loss of fat tissue, and metabolic abnormalities (Mostoslavsky et al., 2006). This severe organismal failure has been associated with defective DNA repair mechanisms, and the observed phenotype resembles a premature aging phenomenon (Mostoslavsky et al., 2006; Toiber et al., 2013).

Furthermore, specific Sirt6 knock-out in the brain is not lethal but causes early growth retardation due to lower growth hormone levels, IGF-1, and learning impairment at 4 months of age (Schwer et al., 2010). These mice resemble early neurodegeneration with tau protein accumulation, increase DNA damage, and cell death, which are hallmarks of neurodegeneration, such as in Alzheimer’s disease (Kaluski et al., 2017). Importantly, acetylated Tau is a pathogenic form of Tau protein, and its accumulation is observed in Alzheimer’s brains. Sirt6 is the main deacetylase that targets this acetylated form of Tau protein (Portillo et al., 2021). In complex brains with a neocortex structure, Sirt6 plays an important role in maintaining its function and its loss causes the onset of premature aging, driving to neurodegeneration.

The loss of Sirt6 expression in a primate model (Cynomolgus Monkeys) leads to perinatal lethality in females and middle gestational death in the only male. This is interesting when comparing c with *D. melanogaste*r lacking a strong phenotype and mouse models that can survive some weeks after birth, both without severe defects in development. In contrast, the primate Sirt6 knock-out model is inviable and presents several developmental defects that can be resumed as a retarded maturation of almost every tissue. These monkeys show severe neurodevelopmental defects, smaller brains, higher levels of immature NPCs, reduced numbers of mature neurons, and abnormal expression of stem cell genes. These results show that Sirt6 is necessary for the proper silencing of stem-associated transcription and the differentiation of NPCs into neurons, as observed before (Zhang et al., 2018). However, it would be interesting to evaluate if Sirt6 loss can also reduce the number of mature neurons by an increased genome instability during the proliferation burst of NSCs and NPCs, which ultimately lead to enhanced cell death.

Importantly, it has been observed that mutation of Sirt6 in humans leads to fetal lethality, and it is incompatible with the proper development of human fetuses. The single-family case reported a homozygous change of a C for a G in the position 187, resulting in a change of aspartic acid for histidine (Asp63His). This mutation affects a highly conserved aspartate residue, which is required for NAD + binding in the catalytic pocket, resulting in a mutant without deacetylase and deacylase activities. Notably, the fetuses presented severe defects in head development, reduced head circumference, and microcephaly, which again points out the role of Sirt6 to ensure the proper development and differentiation of neural cells (Ferrer et al., 2018).

These studies have addressed the effect of Sirt6 in different species, and importantly the loss of Sirt6 increases in severity as the organism presents a more complex brain structure. In protostome organisms, loss of Sirt6 is not incompatible with life, but it affects its lifespan, mainly associated with the functions of Sirt6 regulating metabolism. In mammals, Sirt6 loss has a more severe phenotype; mutant mice can survive for a month with severe growth retardation. When the knock-out is brain-specific, it causes early neurodegeneration. However, in primates such as monkeys and humans, Sirt6 loss is incompatible with life. The monkeys can survive until birth with severe developmental defects, but in humans, it leads to fetal lethality. Also, in primates, the effect of Sirt6 loss in brain development is very evident, causing microcephaly and retardation of brain growth. This phenomenon has been related to the aberrant differentiation of NSCs and NPCs into mature neurons. However, since Sirt6 also functions by maintaining genomic stability, and brain growth requires a proliferative burst, it is also possible that aberrant development in this organ can be due to increased genomic instability in the absence of Sirt6.

## Concluding Remarks

The evolution of the central nervous systems has progressed toward the acquisition of clustered structures of neurons that process information and regulate the organism’s behavioral outcome. As brains become more complex and process higher amounts of environmental information, an increased number of specialized structures and neural cells are required. This phenomenon makes neural cells particularly susceptible to DNA damage since it can arise from an internal source during developmental proliferative bursts. In mature neurons, the absence of proliferation can maintain DNA changes during organismal life. Therefore, during the evolution of mammal brains and especially the human brain, it has been necessary to acquire proteins that maintain neural genomic and epigenomic integrity during the lifespan. In this sense, Sirt6 functions during embryonic and adult neurogenesis, and its role in preventing neurodegeneration during aging led us to suggest that Sirt6 functions as a master regulator of brain function. For example: as a repressor of gene expression, through its epigenetic regulation of histone acetylations, its ability to recruit TF’s and chromatin remodelers, while also preventing the accumulation of DNA damage and the activation of retrotransposable elements. Overall Suggesting that Sirt6 could function as a genomic integrity guardian during the whole life of the organism, and the regulation of Sirt6 during specific time points of organismal life (embryonic development or aging) could have significant results for the function and survival of the organism.

## Future Directions

Sirt6 is an epigenetic regulator of capital importance for the development and proper function of the human brain. As the human brain has become complex, the function of Sirt6 has become more and more necessary during the development and aging of the organ. One of the possible reasons that explain Sirt6 importance is the major role of Sirt6 in the regulation of several DNA repair pathways and the high probability of DNA damage during NPC proliferative burst and neuron metabolic activity. It is necessary to focus specific research lines on the study of physiological levels of DNA damage during neurogenesis and the function of Sirt6 in the proper repair of these lesions. Also, it is essential to study the Sirt6 interactomes and how it has changed during evolution as the brain becomes more and more complex. The study of novel Sirt6 functions and interactions during brain evolution could shed light on the mechanisms that allow Sirt6 to regulate human lifespan, aging phenotype and neurodegeneration.

## Data Availability Statement

The original contributions presented in the study are included in the article/supplementary material, further inquiries can be directed to the corresponding author/s.

## Author Contributions

Both authors contributed with the planning and writing of the manuscript and prepared the figures.

## Conflict of Interest

The authors declare that the research was conducted in the absence of any commercial or financial relationships that could be construed as a potential conflict of interest.

## Publisher’s Note

All claims expressed in this article are solely those of the authors and do not necessarily represent those of their affiliated organizations, or those of the publisher, the editors and the reviewers. Any product that may be evaluated in this article, or claim that may be made by its manufacturer, is not guaranteed or endorsed by the publisher.
